# Case report: Heterotopic pregnancy – A very unlikely cause of haemoperitoneum in trauma setting

**DOI:** 10.1016/j.ijscr.2022.107025

**Published:** 2022-04-02

**Authors:** Phyu Cin Thant, Luis Isabel

**Affiliations:** a Department of Surgery, Royal Darwin Hospital, Northern Territory, Australia; b Royal Darwin Hospital, Northern Territory, Australia

**Keywords:** Case report, Heterotopic pregnancy, Ectopic pregnancy, Haemoperitoneum, Trauma, General surgery

## Abstract

Heterotopic pregnancy is a rare occurrence whereby a concurrent intrauterine and extrauterine pregnancy exists. It is a difficult diagnosis to make early, and often associated with significant morbidity and mortality from complications if diagnosed late. In this report, we present a rare case of heterotopic pregnancy in a 25-year-old woman who presented with abdominal pain following blunt abdominal trauma, and ultrasound showing haemoperitoneum and a viable intrauterine 10-week gestation. CT scan was non diagnostic and significance of left parametrial contrast was not considered. She was admitted under the general surgical team and underwent laparoscopy showing a ruptured left tubal ectopic pregnancy and partial salpingectomy was performed. She recovered well post operatively and has continued to carry the intrauterine pregnancy without complications. Surgeons and emergency physicians should be aware of this condition and to consider it in female patients presenting with acute abdomen or trauma even in the presence of an intrauterine pregnancy.

## Highlights

• Concurrent intrauterine and extrauterine pregnancy is called heterotopic pregnancy.
• Difficult to diagnose early
• Blunt abdominal trauma can rupture the ectopic pregnancy component.
• General surgeons and emergency physicians need to be aware of this diagnosis.

## 1 Introduction

A concurrent intrauterine and extrauterine pregnancy is called heterotopic pregnancy, which occurs in about 1 in 30,000 natural conceptions [1]. The incidence however could be significantly higher up to 1 in 100 with in-vitro fertilisation and other assisted reproductive technologies [1], [2], [3].

Ectopic pregnancies in general are more common occurring in about 2% of pregnancies, with ruptured ectopic pregnancies still accounting for approximately 6% of maternal deaths [4]. Trauma, however, is the leading cause of non-obstetric maternal morbidity and mortality [5]. In the setting of trauma, for a woman with a confirmed intrauterine pregnancy presenting with abdominal pain and haemoperitoneum, non-obstetric causes are considered first [6].

In the following case report, we will discuss the case of a ruptured ectopic pregnancy from trauma causing haemoperitoneum in the setting of a heterotopic pregnancy and how this was managed in an acute surgical unit.

## 2 Case report

**Fig. 1:**
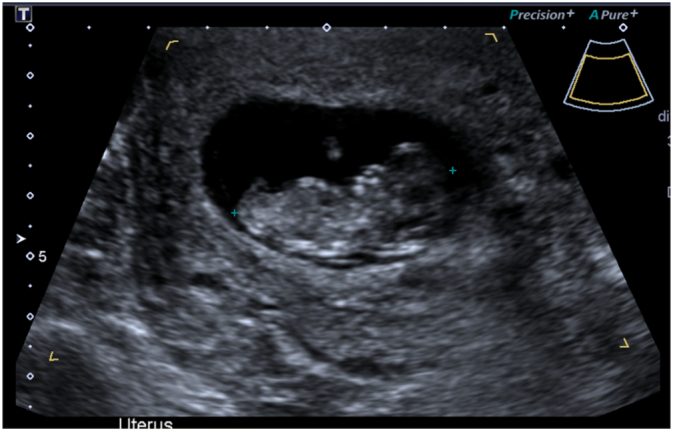
Ultrasound showing live intrauterine foetus.

A 25-year-old Indigenous woman presented to a local health clinic with blunt abdominal trauma following a domestic assault incident. She was kicked on the left side of the abdomen and hit with a pole to the head sustaining a superficial scalp laceration. Other than a history of rheumatic heart disease, she has no history of pelvic inflammatory disease, no previous surgical history and does not take any regular medications. During this visit, she was found to be pregnant based on positive urine beta HCG (G2P1 – previous normal vaginal birth). There was no history of vaginal bleeding nor abdominal pain prior to trauma. She had ongoing left upper quadrant pain the following day, so she was taken to local hospital where abdominal and pelvic ultrasound was done (Fig. 1), showing moderate free fluid in abdominal and pelvic cavity concerning for haemoperitoneum and a single live intrauterine gestation of 10 weeks. Her haemoglobin was noted to be 76 g/L and a unit of blood was given. Following discussion with the surgical team, she was transferred to the tertiary trauma centre.

**Fig. 2:**
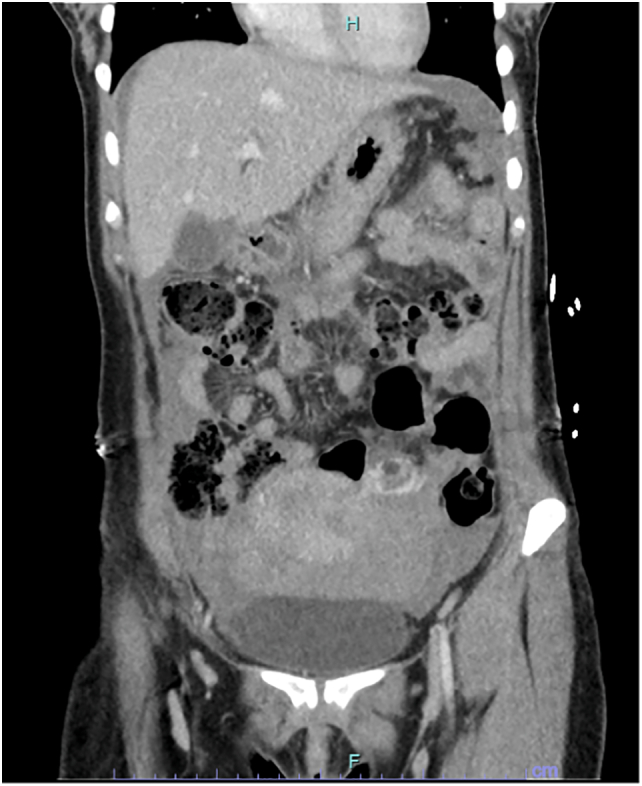
CT scan of the abdomen showing free fluid in the pelvis and upper abdomen.

On arrival to the tertiary hospital, she was stable with a heart rate of 84 and a blood pressure of 102/66 mmHg, with a repeat haemoglobin of 70 g/L despite the transfusion. On examination, her abdomen was soft but generally tender with maximal tenderness in the left upper quadrant. CT head, abdomen, pelvis (portal venous phase only) was done following verbal consent from patient, that showed moderate to large haemoperitoneum throughout peritoneum of uncertain origin (Fig. 2). Left parametrial contrast enhancement was noted of uncertain significance to traumatic presentation.

**Fig. 3:**
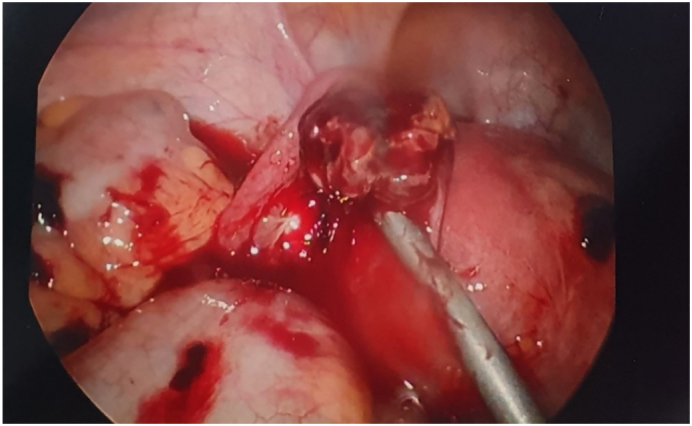
Left ruptured ectopic pregnancy.

She was admitted under the general surgical/trauma team, transfused another unit of packed red cells, and the decision was made to undergo laparoscopy +/− laparotomy and proceed. During the laparoscopy (performed by consultant general surgeon), she was noted to have blood around the liver, spleen and predominantly in left lower quadrant. On further inspection of the left lower quadrant, there was an unexpected finding of a bleeding mass on the left fallopian tube suspicious for a ruptured ectopic pregnancy (Fig. 3). Left partial salpingectomy was performed, haemostasis achieved and approximately 1.2 L of haemoperitoneum was washed out. There was no other intra-abdominal source of bleeding or injury identified.

Post operatively, the patient recovered well and was counselled on the intra-operative finding. The obstetrics and gynecology team were consulted, and a repeat pelvic ultrasound confirmed a viable intrauterine pregnancy. Subsequent histology result confirmed a tubal ectopic pregnancy. The patient was discharged postoperative day 4 with the plan for ongoing outpatient review from the obstetrics team.

## 3 Discussion

Heterotopic pregnancy, which is a simultaneous ectopic and uterine pregnancy, is a rare event. The use of assisted reproductive technology and other risk factors including pelvic inflammatory disease, previous tubal surgery, previous ectopic pregnancy, use of intrauterine device and smoking have shown an increase in the incidence up to 1% [1], [4]. About 70% of heterotopic pregnancies are diagnosed between 5 and 8 weeks of gestation and further 20% between 9 and 10 weeks [2], [7].

Early diagnosis is challenging as most patients are asymptomatic until complications from extrauterine pregnancy arises [1]. This is often a late diagnosis, with associated with significant morbidity and mortality. The reason for late diagnosis is multifactorial - patients normally present with non-specific abdominal pain, imaging with ultrasound can either fail to detect heterotopic pregnancy or be misinterpreted due to presence of an intrauterine pregnancy, and the index of suspicion from the clinician is often low [8]. Particularly with ultrasound imaging, the picture can be mistaken as an intrauterine gestation with a haemorrhagic corpus luteum cyst [7].

In this case, our patient did not have any risk factors as listed above to suggest a concurrent ectopic pregnancy. As this was a trauma presentation where a viable intrauterine pregnancy was confirmed, the differential diagnosis of a ruptured simultaneous ectopic pregnancy was not considered. Due to history of trauma from assault and with her pain being mainly left upper quadrant and haemoperitoneum generally widespread, our differential was a splenic or mesenteric laceration. In terms of radiological imaging, a left parametrial contrast enhancement on CT was commented, but the significance of this was also not considered.

Surgical management with a laparoscopy or laparotomy (depending on the patient's condition) with subsequent salpingotomy/salpingectomy/oophorectomy is the mainstay management of heterotopic pregnancy. It is important to minimise handling of the gravid uterus to provide maximum chance of intrauterine pregnancy developing to full term. Early management has favourable outcomes, with 75% delivering at full term, 16% preterm and just 9% having stillbirth or spontaneous abortion after a laparotomy [6], [7]. With this case, despite requiring blood transfusion, the patient remained haemodynamically normal and therefore, laparoscopy was done as an initial diagnostic tool and bleeding was adequately controlled without requiring conversion to open.

Whilst there has been just one report of a ruptured ectopic pregnancy precipitated by blunt trauma [5], no other case of ruptured ectopic in a heterotopic pregnancy related to trauma has been reported to our knowledge. In this patient, it is highly probable that ectopic component of the heterotopic pregnancy would have ruptured in due course. However, the blunt abdominal trauma has expedited this event.

## 4 Conclusion

It is important for non-obstetric clinicians like surgical and emergency doctors to be aware of this diagnosis. The classical teaching of “suspect ectopic pregnancy in women of child-bearing age until proven otherwise” rings true for female patients presenting with an acute abdomen or haemoperitoneum, even in instances where a viable intrauterine pregnancy has been confirmed. Similarly, abdominal trauma in pregnant women should also be managed as other trauma and thorough examination of intra-abdominal viscera including uterus and fallopian tubes should be performed during exploratory laparoscopy/laparotomy. High index of suspicion and early surgical intervention can minimise the morbidity and mortality of the patient and her intrauterine foetus.

## Sources of funding

None.

## Ethical approval

N/A.

## Consent

Written informed consent was obtained from the patient for publication of this case report and accompanying images. A copy of the written consent is available for review by the Editor-in-Chief of this journal on request.

## Guarantor

Dr. Phyu Cin Thant.

## Registration of research studies

N/A.

The work has been reported in line with the SCARE 2020 criteria [9].

## Provenance and peer review

Not commissioned, externally peer-reviewed.

## CRediT authorship contribution statement

Dr. Phyu Cin Thant – conceptualisation, data curation, writer of original draft.

Dr. Luis Isabel – supervisor, review and editing of the manuscript

## Declaration of competing interest

No conflicts of interest noted.
